# Keeping participants on board: increasing uptake by automated respondent reminders in an Internet-based Chlamydia Screening in the Netherlands

**DOI:** 10.1186/1471-2458-12-176

**Published:** 2012-03-09

**Authors:** Nynke FB Dokkum, Rik H Koekenbier, Ingrid VF van den Broek, Jan EAM van Bergen, Elfi EHG Brouwers, Johannes SA Fennema, Hannelore M Götz, Christian JPA Hoebe, Lydia L Pars, Sander M van Ravesteijn, Eline LM Op de Coul

**Affiliations:** 1 Cluster of Infectious Diseases, Amsterdam Health Service, Amsterdam, The Netherlands; 2 Epidemiology & Surveillance Unit, Centre for Infectious Disease Control, National Institute of Public Health and the Environment, Bilthoven, The Netherlands; 3 STI AIDS Netherlands, Amsterdam, The Netherlands; 4 Department of Infectious Diseases, South Limburg Public Health Service, Geleen, The Netherlands; 5 Department of Infectious Disease Control - division STI/HIV, GGD Rotterdam-Rijnmond, Municipal Public Health Service Rotterdam-Rijnmond, Rotterdam-Rijnmond, The Netherlands

**Keywords:** *Chlamydia trachomatis*, Screening, The Netherlands, Internet

## Abstract

**Background:**

Effectiveness of Chlamydia screening programs is determined by an adequate level of participation and the capturing of high-risk groups. This study aimed to evaluate the contribution of automated reminders by letter, email and short message service (SMS) on package request and sample return in an Internet-based Chlamydia screening among people aged 16 to 29 years in the Netherlands.

**Methods:**

Individuals not responding to the invitation letter received a reminder letter after 1 month. Email- and SMS-reminders were sent to persons who did not return their sample. It was examined to what extent reminders enhanced the response rate (% of package requests) and participation rate (% of sample return). Sociodemographic and behavioural correlates of providing a cell phone number and participation after the reminder(s) were studied by logistic regression models.

**Results:**

Of all respondents (screening round 1: 52,628, round 2: 41,729), 99% provided an email address and 72% a cell phone number. Forty-two percent of all package requests were made after the reminder letter. The proportion of invitees returning a sample increased significantly from 10% to 14% after email/SMS reminders (round 2: from 7% to 10%). Determinants of providing a cell-phone number were younger age (OR in 25-29 year olds versus 16-19 year olds = 0.8, 95%CI 0.8-0.9), non-Dutch (OR in Surinam/Antillean versus Dutch = 1.3, 95%CI 1.2-1.4, Turkish/Moroccan: 1.1, 95%CI 1.0-1.2, Sub Sahara African: 1.5, 95%CI 1.3-1.8, non-Western other 1.1, 95%CI 1.1-1.2), lower educational level (OR in high educational level versus low level = 0.8, 95%CI 0.7-0.9), no condom use during the last contact with a casual partner (OR no condom use versus condom use 1.2, 95%CI 1.1-1.3), younger age at first sexual contact (OR 19 years or older versus younger than 16: 0.7, 95%CI 0.6-0.8). Determinants for requesting a test-package after the reminder letter were male gender (OR female versus male 0.9 95%CI 0.8-0.9), non-Dutch (OR in Surinam/Antillean versus Dutch 1.3, 95%CI 1.2-1.4, Turkish/Moroccan: 1.4, 95%CI 1.3-1.5, Sub Sahara African: 1.4, 95%CI 1.2-1.5, non-Western other: 1.2, 95%CI 1.1-1.2), having a long-term steady partnership (long-term versus short-term.1.2 95%CI 1.1-1.3). Email/SMS reminders seem to have resulted in more men and people aged 25-29 years returning a sample.

**Conclusions:**

Nearly all respondents (99.5%) were reachable by modern communication media. Response and participation rates increased significantly after the reminders. The reminder letters also seemed to result in reaching more people at risk. Incorporation of automated reminders in Internet-based (**C**hlamydia) screening programs is strongly recommended.

## Background

In the Netherlands, a register- and Internet-based Chlamydia Screening Implementation (CSI) started in 2008 in three regions among 16 to 29 year-old sexually active people. The aim of this pilot implementation was to assess whether annual systematic, selective screening can reduce population prevalence of *Chlamydia trachomatis *(*Ct*) and prevent serious complications such as genital and pelvic inflammatory diseases (PID), sub- and infertility and ectopic pregnancies [1]. Feasibility and (cost-)effectiveness of the program were evaluated in order to decide on a national roll-out [2-6].

In systematic screening programs, achieving adequate levels of participation and mobilising high-risk groups are important but challenging [6]. To encourage package request (response) and sample return (participation) in CSI, automated respondent reminders by letter, emails and mobile phone short text message (SMS) were embedded in the screening process. In PILOT CT in 2002-2003 in the Netherlands, it was shown that reminders can contribute up to 18% of the total response [7]. Although the use of reminders has been described in other *Ct *screening programs, reminders were either not sent by modern communication technologies such as email and SMS, their impact on uptake was not extensively evaluated, or they were reported in settings other than systematic population-based screening [8-12]. Email reminders were mostly applied in opportunistic Internet-based *Ct *screenings to remind on checking the test result and obtaining treatment [9,10,13].

In CSI a combination of reminder letters, emails and SMS messages has been used. As part of the process evaluation of the first two screening rounds of CSI we examined (1) the determinants of providing a cell phone number by respondents, (2) to what extent reminders enhanced the response rate (% of package requests) and participation rate (% of sample return), and (3) the determinants for response and participation after a reminder. The outcomes may contribute to the optimisation of screening adherence and the development of - tailored - future population-based screening programs.

## Methods

### Design and procedure CSI

CSI is a systematic population-based Chlamydia screening program with annual screening rounds, using the Internet and home testing kits [3-6]. The target population included all 16 to 29 year old inhabitants of Amsterdam, Rotterdam, and South-Limburg.

**Figure 1:**
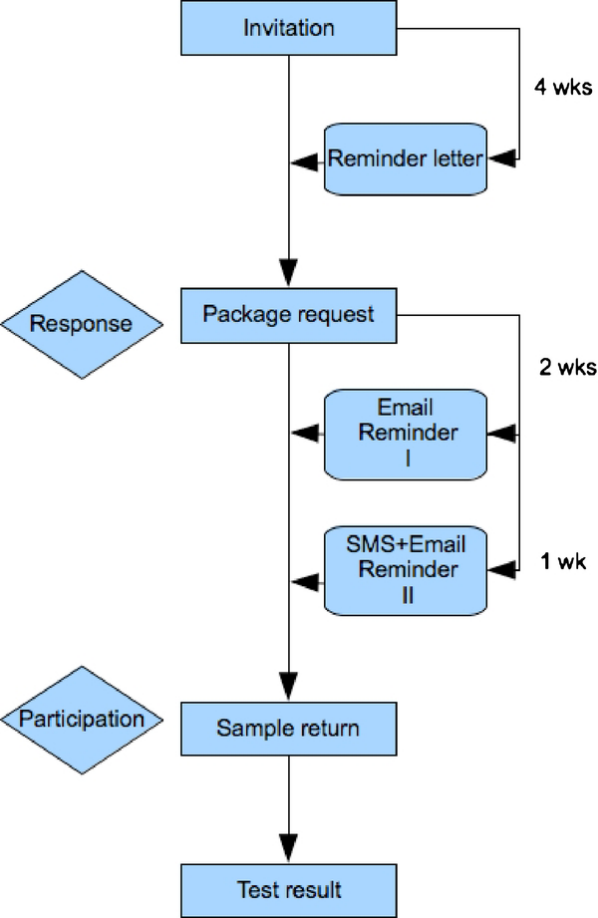
**Flowchart of reminders sent after non-response and non-participation**.

The screening procedure was characterised by five steps: invitation, request of home testing kits, home sampling, sample return, and checking the test result [Figure 1]. Each step was automatically administrated and controlled by a central computer system. Reminders, including a letter, two emails and from 2009 (round 2) an additional SMS, were sent automatically from the screening application (by monitoring barcodes of test packages that were sent out and not returned). First, invitees received an invitation letter with a personal code to log in on http://www.chlamydiatest.nl. Online, invitees could either order a free home testing kit or decline participation in the screening. Respondents were asked to voluntarily provide their email address and cell phone number for communication purposes. Respondents who did not provide an email address or cell phone number (0.5%) did not receive the reminders. Invitees who did not request a test kit within 4 weeks after the initial invitation received a reminder letter by post. After home specimen collection, samples were posted to the regional laboratory. If no sample arrived at the laboratory within 2 to 3 weeks after package sending, the respondent automatically received 1, respectively, 2 email reminder(s). In round 2, the second email was coupled with an SMS. Text messages included a request for sample return. After sample testing at the laboratory, test results were available online using the personal login code. Ct-positives were directed for treatment (general practitioner or STI clinic) and automatically received a re-screening test kit 6 months after their test result. All invitees were invited again in the second screening round (if still fulfilling the age criteria). Screening procedures were similar in both rounds.

In the first round starting in April 2008, 256.400 invitations were sent and in the second round 301.600. Overall response rates (package requests) in the first and second round were 21% and 14% respectively. Participation rates (returned samples by initial invitees) were 16% and 11.5% [3,4] Overall, positivity rates were 4.2% and 4.1% in the first two screening rounds [4].

### Data collection

Sociodemographic data were available for all invitees from population registers. Sexual behavioural data were collected via online questionnaires. Questionnaires were completed voluntarily after invitees replied for a test package. Additional questionnaires were sent to non-participants at day 28 [5,14]. Data collection included age, gender, educational level, sexual history, STI history and symptoms. Process data on invitations, reminders and laboratory results (date and time of sending or receiving) were automatically generated and stored in the screening database. Participation rates were calculated by dividing the number of returned samples by the number of invitations sent. Small differences in numbers could have occurred compared to previous research published [2,4-6] depending on the time of analysis and data completeness, and using various selection criteria for analysis. Subjects with unusual screening procedures, e.g. lost packages and failed tests (3% of the participants in each round) were excluded from the analysis.

### Analyses

The first step was assessing the numbers of respondents providing an email address and/or cell phone number as they were reachable for screening reminders. Proportions of package requests (response rates) after the reminder letter (≥31 days) were compared to response rates without the need of a reminder (≤30 days). Proportions of participation rates (sample return) after the email and SMS reminders (15-30 days) were compared to participation rates without needing a reminder (≤14 days), and to participation rates after a longer period after reminder reception (≥31 days). Since both email - and SMS reminders were sent in a short period, no distinction could be made between these two reminders. Differences between proportions were tested for statistical significance by exact binomial quantities (p-values and overlapping confidence intervals).

Ct-positivity rates were also studied in relation to participation before and after the email/SMS reminders (at 14 days after package delivery).

Univariate and multivariate logistic regression analyses (backwardstepwise, *p <*0.05) were conducted to identify determinants of (1) providing a cell phone number, (2) response rates after a reminder letter, and (3) participation rates after the email/SMS reminders. Determinants for providing an email address were not investigated since 99% of respondents provided their email address.

Variables were included in the multivariate models if *p *≤ 0.20 (Wald test) in univariate analyses (SAS software, version 9.2). Sociodemographic and sexual risk variables (e.g. variables on numbers of partners, condom use, duration of partnerships) were included [6]. Ethnic groups included the main immigrant groups in the Netherlands, based on country of birth of the invitee and the parents from the population register. Community risk level comprised a risk classification of geographic clusters based on age, ethnic and income profiles by neighbourhood [4]. Socio Economic Status (SES) was defined per postal code area as previously described [6].

## Results

### Determinants of providing a cell phone number

Although providing an email address and/or cell phone number was not obligatory in CSI, 99% of all respondents provided an email address (round 1: n = 51,922; round 2: n = 41,172) and 71% a cell phone number in the first screening round and 99%, respectively 73%, in round 2 [Table 1]. Of the respondents, 70% provided both (n = 36,983) in round 1 and 72% (n = 30,170) in round 2). Of all respondents, 0.5% (round 1 n = 314, round 2 n = 217) provided neither an email address nor cell phone number.

**Table 1:** Determinants of providing a cell phone number by responders, per screening round

Package requests (N)	Multivariate logistic regression					
	
	Round 1 (N = 52628)			Round 2 (N = 41729)		
	**n (%)**	**OR (95%CI)**	**p-value**	**n (%)**	**OR (95%CI)**	**p-value**

Cell phone number providers (n)	37,375			30,510		

Gender	37,375			30,510		

Male	33.2	1.0		32.3	NA	NA

Female	68.8	**1.2 **(1.0-1.4)	0.008	67.7		

Age	37,374			30,510		

16-19	14.7	1.0		15.1	1.0	

20-24	39.2	1.0 (1.0-1.1)	0.23	40.4	1.1 (1.0-1.2)	0.08

25-29	46.1	**0.8 **(0.8-0.9)	< 0.001	44.5	**0.8 **(0.8-0.9)	< 0.001

Ethnicity	37,374			30,510		

Dutch	61.2	1.0		60.1	1.0	

Surinam/Antillean	11.8	**1.3 **(1.2-1.4)	< 0.001	11.5	**1.3 **(1.2-1.4)	< 0.001

Turkish/Moroccan	6.4	**1.1 **(1.0-1.2)	0.04	6.3	1.1 (1.0-1.2)	0.09

Sub Sahara African	3.4	**1.5 **(1.3-1.8)	< 0.001	4.1	**1.7 **(1.5-2.0)	< 0.001

Western, other	8.0	1.0 (0.9-1.1)	0.66	8.6	1.0 (0.9-1.1)	0.87

Non-Western, other	9.2	**1.1 **(1.1-1.2)	0.002	9.4	**1.2 **(1.1-1.3)	< 0.001

Region	37,375			30,510		

Rotterdam	57.4	ns	ns	55.0	1.0	

Amsterdam	39.3			38.3	1.0 (0.9-1.0)	0.35

South-Limburg	3.3			6.7	**0.8 **(0.7-0.9)	< 0.001

Community risk level	37,375			30,510		

Low	34.9	1.0		34.4	1.0	

Medium	50.1	**0.9 **(0.8-0.9)	< 0.001	49.0	**0.9 **(0.9-1.0)	0.006

High	15.0	1.0 (0.9-1.0)	0.24	16.6	0.9 (0.9-1.1)	0.82

Educational level	23,229			17,172		

Low	5.0	1.0		4.9	1.0	

Medium	28.7	0.9 (0.8-1.0)	0.17	28.8	1.0 (0.8-1.2)	0.74

High	66.3	**0.8 **(0.7-0.9)	0.007	66.3	**0.8 **(0.7-1.0)	0.05

Ethnicity SP	13,998			9,638		

Concordant(NL/NL)	54.8	1.0		54.1	ns	ns

Discordant (NL/non-NL)	26.9	1.0 (0.9-1.1)	0.55	26.7		

Concordant (non-NL/non-NL)	18.3	**1.4 **(1.2-1.5)	< 0.001	19.2		

Age at first sexual contact	22,728			16,752		

≤5	29.3	1.0		30.6	1.0	

16-18	52.6	**0.8 **(0.8-0.9)	< 0.001	52.9	**0.8 **(0.8-0.9)	< 0.001

≥ 19	18.1	**0.7 **(0.6-0.8)	< 0.001	16.5	**0.6 **(0.6-0.7)	< 0.001

Number of sexual partners < 6 months	22,459			16,612		

No partner(s)	8.5	1.0		7.9	ns	ns

1 steady partner	49.4	1.2 (1.0-1.5)	0.12	44.5		

1 casual partner	11.7	1.2 (1.0-1.5)	0.16	13.0		

≥ 2 partners (incl. steady partner)	30.4	**1.5 **(1.1-1.9)	0.003	34.6		

Duration steady partnership	22,812			16,841		

< 1 year	15.9	1.0		17.7	1.0	

1-2 years	20.9	1.0 (0.9-1.1)	0.62	19.5	1.0 (0.9-1.1)	0.18

3-5 years	15.0	**0.8 **(0.7-0.9)	< 0.001	12.7	**0.9 **(0.8-1.0)	0.01

≥ 6 years	9.1	**0.8 **(0.7-0.8)	< 0.001	6.9	**0.7 **(0.6-0.8)	< 0.001

No steady partner	39.1	1.5 (0.9-2.4)	0.13	43.2	**0.8 **(0.7-0.9)	0.001

Condom use last contact CP	8,124			6,926		

Yes	47.7	1.0		44.0	1.0	

No	52.3	**1.2 **(1.1-1.3)	< 0.001	56.0	**1.1 **(1.1-1.3)	0.02

Condom use last contact SP	13,876			9,547		

Yes	15.8	1.0		16.0	NA	NA

No	84.2	**1.2 **(1.1-1.3)	< 0.001	84.0		

History of STI	3,175			8,442		

No STI ever	68.1	1.0		68.8	1.0	

Yes, < 6 months	4.0	1.2 (0.8-2.0)	0.38	4.0	1.2 (0.9-1.5)	0.25

Yes, ≥ 6 months	27.9	**1.2 **(1.0-1.5)	0.03	27.2	**1.2 **(1.1-1.3)	0.002

Symptoms of an STI	5,649			13,284		

Yes	38.1	ns	ns	40.1	1.0	

No	61.9			59.9	**0.7 **(0.8-0.9)	< 0.001

OR, odds ratio; CI, confidence interval; NA, not applicable (*p *≥ 0.2 in univariate analysis); ns, not significant (*p *> 0.05 in multivariate analysis); MSM, men having sex with men, STI, sexually transmitted infection(s); SP, steady partner; CP, casual partner. Ethnicity 'Western other' included Oceania, North America, Canada, Europe (excluding the Netherlands and Turkey); 'Non Western other' included Central and South America, Middle East, South and South-East Asia (excluding Surinam, Antilles, Aruba, Morocco and North Africa). Non-significant variables in the multivariate models in both screening rounds are not shown: SES-score, living situation, concurrent partners, sexual preference, history of STI testing

Sociodemographic factors that were positively associated with providing a cell phone number in both screening rounds were younger age (OR in 25-29 year olds versus 16-19 year olds = 0.8, 95%CI 0.8-0.9), non-Dutch (OR in Surinam/Antillean versus Dutch = 1.3, 95%CI 1.2-1.4, Turkish/Moroccan: 1.1, 95%CI 1.0-1.2, Sub Sahara African: 1.5, 95%CI 1.3-1.8, non-Western other 1.1, 95%CI 1.1-1.2), lower educational level (OR in high educational level versus low level = 0.8, 95%CI 0.7-0.9). Some sexual behavioural risk factors showed small but significant associations OR's between 1.1-1.5) with providing a cell phone number: young age at first sexual contact (≤15 years), having a short-term steady partnership (≤1-2 years), no condom use during last sexual contact with a casual partner, and having hadan STI more than 6 months ago [Table 1]. Characteristics of persons providing a cell phone number that were only significant in one of the two screening rounds were female gender, living in a main city, being in a concordant non-Dutch/non-Dutch steady partnership, having ≥ 2 sexual partners in the past 6 months, no condom use during the last sex contact with a steady partner, and being *Ct*-positive in the first screening round.

### Effect of automated reminders on response- and participation rates

**Figure 2:**
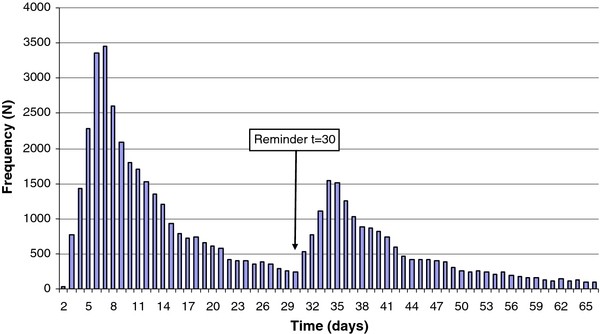
**Time (days) from invitation to test package request in the first screening round**. Update (1 year data) from previously published graph (3 month data) by van Bergen et al. BMC infectious diseases 2010, 10:293.

Of all invitees, 87% (223,700/256,400) and 91% (275,000/301,600) received a reminder letter in the first, respectively second, screening round. Of people who requested a test package (± 52,600 in round 1 and ± 41,700 in round 2), 41% and 43% did their request after the reminder letter. The overall response rate in the first round increased significantly (p < 0.0001) from 12% to 20% (70% increase) after the reminder letter and in round 2 from 8% to 14% (75% increase) as shown in Figure 2. Request patterns were similar in both screening rounds (round 2 not shown).

### Determinants of response after the reminder letter

Factors that were significantly associated with requesting a test package after receiving a reminder letter in both screening rounds were male gender (OR female versus male 0.9 95%CI 0.8-0.9), non-Dutch background (OR in Surinam/Antillean versus Dutch 1.3, 95%CI 1.2-1.4, Turkish/Moroccan: 1.4, 95%CI 1.3-1.5, Sub Sahara African: 1.4, 95%CI 1.2-1.5, non-Western other: 1.2, 95%CI 1.1-1.2), having a long-term steady relationship (long-term versus short-term.1.2 95%CI 1.1-1.3) [Table 2]. In round 1, responding after the reminder letter was also associated with young age (16-19 yrs), having had a casual partner or 2 or more sex partners in the past 6 months, and being a man with a heterosexual preference (compared to men who have sex with men (MSM)). People who tested *Ct*-positive in round 1 and did not return the re-screening test kit that was sent after 6 months, were more likely to respond after the reminder letter in round 2, compared to *Ct*-negatives and *Ct*-positives who did return the re-screening sample.

**Table 2:** Determinants of package request after the reminder letter, per screening round Multivariate logistic regression

	Multivariate logistic regression			
	
	Round 1 (n = 20926)		Round 2 (n = 17267)	
	
	OR (95%CI)	p-value	OR (95%CI)	p-value
Gender				
Male	1.0		1.0	
Female	**0.9**(0.8-0.9)	< 0.001	**0.9**(0.8-0.9)	< 0.0001
Age				
16-19	1.0		ns	ns
20-24	**0.9**(0.9-1.0)	< 0.001		
25-29	**0.9**(0.8-0.9)	< 0.001		
Ethnicity				
Dutch	1.0		1.0	
Surinam/Antillean	**1.3**(1.2-1.4)	< 0.001	**1.2**(1.1-1.3)	< 0.001
Turkish/Moroccan	**1.4**(1.3-1.5)	< 0.001	**1.4**(1.2-1.5)	< 0.001
Sub Sahara African	**1.4**(1.1-1.3)	< 0.001	**1.4**(1.2-1.5)	< 0.001
Western, other	**1.2 **(1.1-1.3)	< 0.001	**1.1**(1.0-1.2)	0.02
Non-Western, other	**1.2**(1.1-1.2)	< 0.001	**1.2**(1.1-1.3)	< 0.001
Region				
Rotterdam	1.0		1.0	
Amsterdam	**1.3**(1.2-1.3)	< 0.001	**1.2**(1.1-1.2)	< 0.001
South-Limburg	**1.3**(1.1-1.4)	< 0.001	1.0 (0.9-1.1)	0.61
Number of sexual partners < 6 months				
No partner(s)	1.0		ns	ns
1steady partner	**1.8**(1.4-2.3)	< 0.001		
1 casual partner	**2.2**(1.7-2.8)	< 0.001		
≥ 2 partners (steady partner included)	**2.0**(1.6-2.6)	< 0.001		
Duration steady partnership				
< 1 year	1.0		1.0	
1-2 years	1.0(1.0-1.1)	0.27	1.1(1.0-1.2)	0.05
3-5 years	1.0(0.9-1.1)	0.87	**1.3**(1.1-1.4)	< 0.001
≥ 6 years	**1.2**(1.1-1.3)	0.009	**1.3**(1.2-1.5)	< 0.001
No steady partnership	1.0(0.9-1.1)	0.92	**1.2**(1.1-1.3)	0.001
Condom use last contact CP				
Yes	1.0		1.0	
No	**0.9**(0.8-1.0)	0.004	**0.9**(0.8-1.0)	0.004
Sexual preference				
Heterosexual mean	1.0		ns	ns
MSM	**0.8**(0.6-0.9)	0.003		
Test result first screening round				
Ct-neg	NA	NA	1.0	
Ct-pos, no rescreening			**1.9(**1.2-3.1)	0.01
Ct-pos, rescreened Ct-neg			1.1(0.9-1.4)	0.19
Ct-pos, rescreened Ct-pos			0.6(0.3-1.5)	0.30

OR, odds ratio; CI, confidence interval; NA, not applicable (*p*≥ 0.2 in univariate analysis); ns, not significant (*p *> 0.05 in multivariate analysis); MSM, men having sex with men, STI, sexually transmitted infection(s); SP, steady partner; CP, casual partner. 'Western other' included Oceania, North America, Canada, Europe (excluding the Netherlands and Turkey); 'Non Western other' included Central and South America, Middle East, South and South-East Asia (excluding Surinam, Antilles, Aruba, Morocco and North Africa). Non-significant in the multivariate models in both screening rounds are not shown: SES-score, educational level, living situation, ethnicity SP, age at first sex contact, concurrent partners, condom use with SP, STI history. Non-significant variables in second round only: gender, age, number of sexual partners < 6 months, sexual preference

Both models have been studied by gender. However, effects were either smaller or non significant due to smaller numbers (especially in the model for men). These analyses did not provide additional insights and were therefore not presented.

### Participation rates after email- and SMS reminders

Of all responders, 79% (41,700/52,600) in round 1 and 82% (34,200/41,700) in round 2 returned a sample to the laboratory. Two weeks after package delivery, reminder emails were sent to half of the responders (round 1: 49% and round 2: 48%), because they did not return their sample. After another week a second reminder was sent to 41% (round 1) and 35% (round 2) of the responders who didn't return their sample.

**Figure 3:**
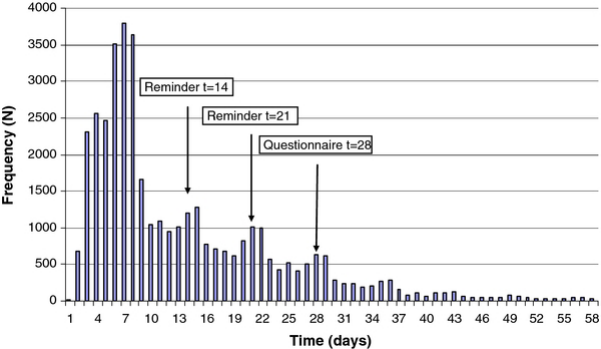
**Time (days) to return test sample after package receipt in the first screening round**.

In the period after the email and SMS reminder were sent, sample return seem to have increased (Figure 3). Of all samples returned, 65% and 64% was returned before any reminder; an additional 26% and 27% after ≥ 1 reminders (day 15-30). After ≥ 31 days, another 9% returned their sample. On day 28, a questionnaire was sent to responders that did not return a sample to ask for their reasons. This may have had a small additional effect (Figure 3). In total, participation rates (proportion of initial invitees returning a sample) increased significantly from 10% to 14% after the email/SMS reminders in round 1 and in round 2 from 7% to 10%. Similar patterns were seen in both screening rounds (round 2 not shown).

### Determinants of participation after email- and SMS reminders

Sociodemographic determinants of participation (sample return) after email/SMS reminders included male gender and persons aged 25 to 29 years. No significant associations were found with sexual behavioural factors; effects were either small or not present in both screening rounds (not shown).

### Chlamydia-positivity

Of all *Ct*-positives in CSI, 67% returned their sample without receiving any reminder and 23% after an email/SMS reminder. Positivity rates among participants that returned their sample before a reminder were 4.5% (round 1) and 4.1% (round 2) and 3.6% among those who sent it after reception of the first 14-day-email/SMS reminders (in both rounds). In the subgroup who sent it back after a longer period (≥ 31 days, including a second reminder on day 21 and the questionnaire on day 28) the positivity rates were not significantly different;4.3% and 4.6%.

## Discussion

Response - and participation rates increased significantly after letter-, email- and SMS reminders were sent in the *Ct*-screening among 16 to 29 year old people in the Netherlands. The reminder invitation letter may not only resulted in more test package requests, but may have also resulted in reaching more people at higher risk of a chlamydia infection (sub-Saharan Africans and Surinamese/Antilleans, persons with a casual partner or ≥ 2 partners in the past 6 months, and *Ct*-positives who did not participate in the re-screening after 6 months). Being at higher risk and fear for the test result may have played a role in being reluctant to request a package at the initial invitation. The reminder letter could have raised more awareness on the importance of being tested for chlamydia. In a previous study [14] it was shown that *Ct*-positives who did not participate in the re-screening after 6 months are at highest risk for a chlamydia infection in the second screening round, compared to *Ct*-negatives and *Ct*-positives who participated in the re-screening. This underlines the importance of reminders to reach this particular high risk group.

In contrast, people with a long-term steady relationship also responded more often after a reminder. Possibly, this group did not respond at first due to a perceived low risk. However, a reminder letter addressing the relevance of screening could have made them decide differently.

Compared to heterosexual men, MSM more often applied for a test package directly after the invitation, which can be related to familiarity with Internet-based interventions targeted to MSM in the Netherlands [15]. Men in general were more likely to react after reminders than women, an effect that was also seen in PILOT CT [7]. Although differences were small, this might be explained by the fact that participation in population-based *Ct*-screening programs in general is higher in women than in men [8,9,12,16,17]. In contrast, in France, women's reaction to reminders (phone call and letters) was little higher than men's, but also in this screening program women participated more often then men [12]. Additionally, in the UK, young women (16-24 yrs) with the highest *Ct*-prevalence participated only after repeated reminders (by postcard, letter or phone call). In this study, again, more women participated than men [8,18]. Concluding, reaching men with reminders is encouraging, but still they are less likely to engage in *Ct*-screenings.

Participation rates increased from 10% to 14% and 7% to 10% in the first respectively second screening round after email/SMS reminders were sent. Still, every 1 in 5 packages was never returned, even after several reminders. Reasons for non-participation (no sample return after package request) were described previously [2,19]. The most important reasons were lack of time, loss of package, forgetfulness and being *Ct*-tested and -treated elsewhere.

Furthermore, we observed that the respondents' willingness to provide an email address or cell phone number for communication during the screening procedure was very high. Therefore, nearly all respondents were reachable by modern communication media (99% by email; 72% by cell phone). In contrast to package request, sample return after email/SMS reminders was not significantly related to (sexual) risk factors of *Ct-*positivity. However, 23% of all *Ct*-positives returned their sample after receiving an email/SMS reminder which illustrates the usefulness of automated reminders to encourage sample return and, subsequently, treating more people for their infection.

Although these findings are encouraging, the magnitude of the effect of email/SMS reminders remains uncertain due to not knowing what proportion of people would have returned their sample eventually without the necessity of receiving a reminder. Assuming that the natural trend of returning a sample would be gradual decline (trend without peaks, Figures 2 and 3), the peaks may represent the additional effect of the reminders, as they immediately follow after the reminders were sent.

We also showed that providing a cell-phone number was slightly associated with risk factors of *Ct-*positivity (ORs up to 1.7). Menon-Johansson et al. reported similar characteristics of people being both at higher risk of STI and more often making use of mobile phones (young aged, migrants, and lower SES) [20].These findings illustrate the value of using cell phones and text message (SMS) for communication to high-risk demographic groups.

To our knowledge CSI is the first systematic, population-based *Ct*-screening program using a combination of reminder letters, emails and SMS to enhance response- and participation rates. Generally, the only systematic [8] and other opportunistic *Ct*-screening programs implemented reminders for similar and other purposes than in CSI. Reminders were used to confirm package receipt [8,12,18], encourage sample return [8,11,12,17,18], checking test results [9,10] or reminding going for treatment [9]. Although reminders were reported in those programs, comparison was impeded due to differences in program design and reminder implementation and the lack of detailed evaluations. Some screening programs reported using modern technologies like email and SMS as reminders. Emails were used to remind on checking test results and getting treatment [9,10], but SMS reminding was reported only once in a small study [17]. More frequently SMS was used for partner- and test result notification [16,17,20-22]. The Internet-based set up of our screening program and the detailed automated monitoring of each logistic step in the screening process made it possible to thoroughly evaluate the effects and determinants of the various reminders. Another great advantage of CSI was the availability of sociodemographic data for all invitees from population registers. One limitation of our evaluation was that due to the program design (implementation), no control group of persons who did not receive reminders was embedded, which makes it more difficult to relate the additional effect to the reminders. On the other hand, the high peaks in response right after the reminders as shown in the graphs are likely showing the contributions of the reminders.

## Conclusion

The results of this evaluation illustrated that implementation of respondent reminders may be effective in increasing response- and participation rates in population-based *Ct-*screening programs. In particular, reminder letters are effective to stimulate package request with eligible, initially not involved, target groups. In CSI, the reminder letter nearly doubled package request rates and seem to have resulted in reaching more people at higher risk. Using email **- **and SMS reminders in *Ct*-screenings is recommended to encourage participation and easily reach young, high risk, target populations, but also other screening programs might benefit from email and SMS reminders. Those providing a cell phone number were at slightly higher *Ct*-risk, which provides extra opportunities to reach these groups.

## Competing interests

The authors declare that they have no competing interests.

The evaluation of the Chlamydia Screening Implementation is being carried out by request of the Ministry of Health, Welfare and Sport, and is funded within the STI programme at the Centre for Infectious Disease Control (grant V/210261/01/IE 'Interventie en Evaluatieonderzoek').

The screening programme and evaluation have been approved by a Medical Ethics Committee of the VUmc in Amsterdam (METc number: 2007/239). All participants provided online informed consent.

## Authors' contributions

ND, RK and EC analysed and interpreted the data, and drafted the manuscript. Other authors were involved in the roll-out of the programme (including data acquisition) and contributed to drafting and revision of the paper. JB and LP were responsible for the coordination of the screening in the three regions; CH and EB, for the screening in South Limburg, JF and RK in Amsterdam, and HG and SR in Rotterdam. IB is involved in the evaluation of the program. CSI project group: JEAM van Bergen, IVF van den Broek, EEHG Brouwers, JSA Fennema, HM Götz, CJPA Hoebe, RH Koekenbier, ELM Op de Coul, LL Pars, SM van Ravesteijn. All authors read and approved the final manuscript.

## Pre-publication history

The pre-publication history for this paper can be accessed here:

http://www.biomedcentral.com/1471-2458/12/176/prepub
